# Ocular Lesions Related to COVID‐19 and Its Vaccines

**DOI:** 10.1155/joph/7078264

**Published:** 2025-12-11

**Authors:** Tao Liu, Mengyao Li, Lin Zhu, Ruyu Liang, Peng Zhang, Xiaoli Liu

**Affiliations:** ^1^ Ophthalmologic Center of the Second Hospital, Jilin University, Ziqiang Street 218, Changchun, China, jlu.edu.cn; ^2^ Radiology Department of the Second Hospital, Jilin University, Ziqiang Street 218, Changchun, China, jlu.edu.cn

**Keywords:** conjunctivitis, COVID-19, eye, retinopathy, uveitis

## Abstract

**Objective:**

To review COVID‐19 infection and COVID‐19 vaccine‐related ocular lesions.

**Methods:**

We carried out a systematic search in PubMed, Web of Science, Embase, and the Cochrane Library on COVID‐19 and ophthalmology and reviewed the incidence, specific manifestations, and risk factors for COVID‐19‐related eye diseases and the relationship between the detection of COVID‐19 in the conjunctiva and tears and eye involvement.

**Results:**

Conjunctivitis was the most common ocular lesion caused by 2019‐nCoV infection, followed by uveitis and retinopathy. Conjunctivitis can be the first manifestation of COVID‐19 infection and may be clinically related to the severity of pneumonia caused by COVID‐19. In particular, conjunctivitis that occurs after pneumonia suggests that the patient has severe systemic disease. COVID‐19 infection can cause uveitis, but the infection rate of COVID‐19 in patients with uveitis is similar to that of the general population. Patients with uveitis need to reduce the dosage of systemic hormones and discontinue biological agents after being infected with COVID‐19. Retinopathy caused by COVID‐19 infection is mainly manifested as retinal microvascular disease, and the prognosis is good. SARS‐CoV‐2 detection in the conjunctiva and tears has high sensitivity and is of great value for disease diagnosis. Eye lesions caused by the COVID‐19 vaccine, similar to other vaccines, have a low incidence and a good prognosis.

**Conclusion:**

COVID‐19‐related ocular lesions are mainly manifested as conjunctivitis, uveitis, and retinal microvascular changes. These diseases are somewhat self‐limiting and have a good prognosis.

## 1. Introduction

SARS‐CoV‐2 is a highly infectious respiratory RNA virus that belongs to the *Coronaviridae* family and the *Betacoronavirus* genus. Coronavirus (SARS‐CoV), which caused SARS in 2003, and MERS‐CoV, which causes Middle East respiratory syndrome, are also in the coronavirus genus. SARS‐CoV‐2 spreads mainly through droplets, aerosols, and contact. It then binds to angiotensin‐converting enzyme II (ACE2) receptors in the lungs, intestines, kidneys, and other tissues, causing varying degrees of acute upper and/or lower respiratory syndrome, as well as symptoms [1–4]. The impact of COVID‐19 on the eyes has attracted our attention. Its direct infection of the eye surface may lead to conjunctivitis, and the cytokine storm caused by COVID‐19 may lead to uveitis and other autoimmune eye diseases. COVID‐19 also causes blood hypercoagulability, which may result in thrombosis, including retinal vein and artery thrombosis. Since the COVID‐19 pandemic, the widespread use of steroids has caused a decline in immune status, leading to the occurrence of rhino‐orbital‐cerebral mucormycosis (ROCM), which has a high mortality rate. The changes in immune status caused by the COVID‐19 vaccine may lead to the onset of uveitis, and vaccine‐induced immune thrombocytopenia (VITT) predisposes individuals to thrombus formation [5–8].

There are SARS‐CoV‐2 action sites in the eye, including ACE2 and transmembrane serine protease (TMPRSS2). ACE2 is the key functional receptor of SARS‐CoV‐2, binding to its spike protein and leading to the endocytosis of the virus [9]. TMPRSS2 helps SARS‐CoV‐2 bind to ACE2 and enter cells by activating the spike protein of the virus [10]. ACE2 receptors are expressed in eye tissues, including the conjunctiva, cornea, aqueous humor, ciliary body, choroid, and retina [11, 12]. TMPRSS2 is also expressed in the conjunctiva and cornea of the ocular surface [1]. Although ACE2 and TMPRSS2 are expressed at low levels in ocular tissues, they provide a potential pathway for SARS‐CoV‐2 to invade the eyes.

The COVID‐19 vaccine is widely administered around the world to reduce the spread of COVID‐19. Some new or recurrent eye diseases have been observed after COVID‐19 vaccination. This article aims to aid in clinical diagnosis and differential diagnosis by reviewing the existing literature. It summarizes the clinical features of eye diseases associated with both COVID‐19 infection and COVID‐19 vaccines.

## 2. Methods and Subjects

**Figure 1:**
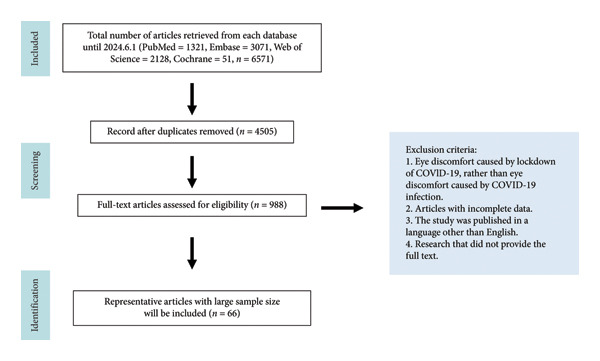
Flowchart of the included studies.

We searched articles published before June 2024 on PubMed, Embase, Web of Science, and Cochrane. The search query used to search for articles included a combination of terms for eye manifestations and diseases and various terms related to SARS‐CoV‐2 and COVID‐19, as well as vaccines. The search included terms such as “Eye Manifestations,” “SARS‐CoV‐2,” “COVID‐19 Virus,” “COVID‐19 Vaccines,” and many others. Free words were obtained by searching in PubMed’s MeSH vocabulary. A total of 6571 records were retrieved from the four databases (PubMed, Embase, Web of Science, and Cochrane) using this search formula. After removing duplicates, the full texts of the remaining 4505 articles were reviewed. Literature was excluded if it met any of the following criteria: (1) Eye discomfort caused by the COVID‐19 lockdown, rather than by COVID‐19 infection. For example, eye discomfort due to prolonged use of electronic devices during the pandemic. (2) Articles with incomplete data. (3) The study was published in a non‐English or non‐Chinese language, or the full text was unavailable. Guiding and landmark “to editor” articles and related articles were also included. After these screenings, 66 articles were finally included. This study complies with the Declaration of Helsinki and was approved by the Ethics Committee of the Second Hospital of Jilin University (Figure 1).

## 3. Results

COVID‐19 can cause various ocular manifestations, primarily including conjunctival congestion, dry eyes, epiphora, itching, foreign body sensation, eye pain, and burning. Nasiri summarized the ocular manifestations of 8216 COVID‐19 patients worldwide from December 2019 to August 2021 in a meta‐analysis [13]. The incidence of ocular manifestations related to COVID‐19 was approximately 11.03%. The most common eye manifestation was dry eye or foreign body sensation (16%, *n* = 138), followed by redness (13.3%, *n* = 114), tearing (12.8%, *n* = 111), itching (12.6%, *n* = 109), eye pain (9.6%, *n* = 83), and discharge (8.8%, *n* = 76). Other studies also suggest that conjunctival congestion is the most common ocular manifestation of COVID‐19 [4, 14–18].

It is controversial whether eye manifestations in COVID‐19‐infected patients are related to the severity of the disease. Some studies suggest that patients with severe COVID‐19 are more likely to have eye manifestations [19–21]. Zhong et al. analyzed the characteristics of ocular manifestations related to COVID‐19 in a meta‐analysis [4]. They summarized 30 studies (5717 patients with COVID‐19) published before June 2021. The most common ocular manifestation was conjunctival congestion (7.6%), followed by epiphora (6.9%), foreign body sensation (6.9%), and conjunctival secretion (4.8%). The incidence of eye diseases was higher in patients with severe COVID‐19, with an odds ratio (OR) of 2.77. However, some studies suggest that eye manifestations in COVID‐19 are not related to the severity of the disease. In fact, they are even more commonly observed in patients with mild COVID‐19 [22]. More research is needed to determine whether ocular manifestations are more likely to occur in severe patients.

### 3.1. Anterior Segment Manifestations

#### 3.1.1. Conjunctivitis

Conjunctivitis was the most common ocular manifestation of COVID‐19 that may be one of the signs of COVID‐19 invading the eyes [23]. The incidence of conjunctivitis in COVID‐19 patients varies from 0.9% to 36% across different studies [24]. This difference may be related to the varying SARS‐CoV‐2 strains prevalent in different regions, medical levels, and patient conditions. Guemes‐Villahoz et al. analyzed 301 COVID‐19 patients (35 diagnosed with conjunctivitis) hospitalized in Madrid, Spain, in 2020 and found that compared with other viral conjunctivitis types, COVID‐19 conjunctivitis has some unique clinical manifestations; Adenoviral conjunctivitis (54.29%) is mostly unilateral, while COVID‐19‐associated conjunctivitis is mostly bilateral. Adenoviral conjunctivitis tends to worsen in the first few days and may last for more than 14 days. The onset time of COVID‐19 conjunctivitis is not fixed (the median onset time is 3 days). It can not only be the first symptom of COVID‐19 but also occur in the middle and late stages of the disease [25].

Conjunctivitis caused by COVID‐19 infection is self‐limiting. Shen found 28 conjunctivitis patients out of 3198 patients hospitalized for COVID‐19 in China in 2021; 15 patients took levofloxacin, 9 patients did not take any treatment, 2 patients took ganciclovir, and 2 patients took artificial tears and other treatments. All conjunctivitis patients had a good prognosis [26]. Spanish studies also suggest that COVID‐19‐associated conjunctivitis is a self‐limiting disease [25]. Conjunctivitis can be the first manifestation of COVID‐19, and its occurrence in the late stage may indicate that the systemic disease is serious [27]. It is mostly a self‐limiting disease with a good prognosis [25].

### 3.2. Posterior Segment Manifestations

#### 3.2.1. Uveitis

**Figure 2:**
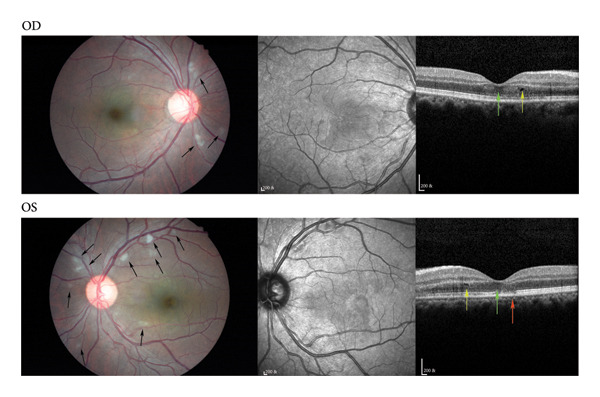
Optical coherence tomography of a patient with white cotton wool patches after COVID‐19 infection. A 43‐year‐old male patient had declined vision after a 3‐day infection with COVID‐19. His vision was 0.04 and 0.02 in the right and left eyes, respectively. Fundus pictures showed white cotton wool patches scattered (black arrow) along the vascular arch of both eyes. Optical coherence tomography showed macular edema (yellow arrow), swelling of the outer retina (green arrow), and discontinuity of the ellipsoidal band (red arrow) (OD: right eye, OS: left eye).

Immune system changes in COVID‐19 patients are closely related to the occurrence of uveitis. The mechanism of uveitis may involve the uncontrolled systemic inflammatory reaction caused by bystander activation, molecular simulation, and direct local virus invasion after COVID‐19 infection [28]. Therefore, the infection rate of COVID‐19 in patients with uveitis and whether COVID‐19 causes uveitis need further investigation. Miller analyzed medical insurance claim data in the United States from January to December 2020. They found that the infection and hospitalization rates of COVID‐19 in 29,869 patients with noninfectious uveitis were not higher compared to 5,776,358 controls without noninfectious uveitis. The use of systemic corticosteroids was significantly associated with an increased risk of COVID‐19 infection and hospitalization [29]. Studies have shown that proinflammatory cytokines in patients with severe COVID‐19 infection are significantly upregulated, which is called “cytokine storm syndrome,” in which interleukin‐6 (IL‐6) and tumor necrosis factor‐α (TNF‐α) play a major role. The use of biological agents and glucocorticoids will reduce the content of related cytokines, thus reducing the hospitalization rate [30]. Moreover, 139 global uveitis experts suggested that after the diagnosis of COVID‐19 in patients with uveitis, the use of biological agents should be stopped, and the use of glucocorticoids should be reduced slowly [31]. Although the Arab study found that the decrease in the hospitalization rate of uveitis may be related to the use of biological agents, the use of biological agents will reduce the body’s resistance and increase the possibility of COVID‐19 replication. Therefore, experts recommend that patients with uveitis stop using biological agents after the diagnosis of COVID‐19 (Figure 2).

COVID‐19 infection can lead to the recurrence or onset of uveitis. The incidence of uveitis is low. In a study conducted in the Arab region, two patients developed recurrent uveitis after discontinuing the use of biological agents. This may be related to the discontinuation of biological agents, but it cannot be ruled out that COVID‐19 infection causes uveitis [32]. In addition, there have been cases of new uveitis after COVID‐19 infection. Francois et al. reported a case of COVID‐19‐associated panuveitis presenting as optic neuropathy in France in March 2020. A 50‐year‐old woman was admitted due to severe bilateral pneumonia. Her throat swab was positive, and she had no previous history of related eye disease. On the second day after admission, she developed blurred vision, redness in her right eye, and eye rotation pain. She was diagnosed with panuveitis‐related ophthalmopathy caused by COVID‐19 [33]. Benito‐Pascual et al. reported that a 60‐year‐old woman without a previous history of eye pain, blurred vision, and a red left eye (OS) was in emergency treatment in August 2020. Imaging examination showed panuveitis and optic neuritis. Ten days later, she developed a dry cough and dyspnea. The polymerase chain reaction (PCR) of COVID‐19 was positive in the nasopharyngeal exudate. She was diagnosed with COVID‐19 complicated by panuveitis and optic neuritis [34].

In general, the infection rate of COVID‐19 in patients with uveitis does not increase, and the hospitalization rate after COVID‐19 infection is even lower [29, 30]. Patients with COVID‐19 infection should stop using biological agents immediately and reduce the use of glucocorticoids [31]. After the diagnosis of COVID‐19, both uveitis recurrence and new onset occur, but the incidence rate is low, and the mechanism is unknown [32–34].

#### 3.2.2. Changes of Retina and Choroid Associated With COVID‐19

COVID‐19 infection can also cause retinopathy. The risk of retinal microangiopathy in patients with COVID‐19 was 8.86 times higher than in those without COVID‐19 [35, 36]. Ivernizzi et al. analyzed the fundus conditions of 32 patients (59 eyes) with confirmed COVID‐19 in Italy during May 2020. The analysis included fundus photos taken within 1 month after the onset of COVID‐19 symptoms and follow‐up photos taken approximately 6 months (±30 days) after the first fundus photography. These patients had no previous history of eye disease. They found that at the initial stage, 12 eyes (20.3%) had retinal hemorrhage, 2 eyes (3.4%) had cotton wool spots, 12 eyes (20.3%) had venous dilatation, and 10 eyes (16.9%) had venous tortuosity. At the follow‐up, only 1 eye (1.7%) had retinal hemorrhage, 4 eyes (6.8%) had varicose veins, and 4 eyes (6.8%) had varicose veins. We also found that after infection with COVID‐19, some patients had white cotton wool patches because of retinal capillary artery occlusion. At follow‐up, the fundus findings related to COVID‐19 showed significant improvement. It is noteworthy that in nonsevere cases, there was no significant difference in the mean artery diameter (MAD) and mean vein diameter (MVD) between the follow‐up group and the control group without COVID‐19 infection. However, in severe cases, there was still a significant difference in MAD and MVD between the follow‐up group and the control group without COVID‐19 infection. This suggests that the changes in the retinal vascular system in nonsevere cases will subside with the passage of time, but serious cases cause damage to the vascular system and may form long‐term continuous changes. This theory is also supported by a pathological finding of retinal vascular wall remodeling in patients who died of COVID‐19 [37, 38].

In addition, some studies have found changes in retinal macular vascular density (VD) related to COVID‐19 [37, 38]. From December 2020 to May 2021, Gündoğan et al. measured the average retinal vascular thickness of 30 eyes of 30 patients with severe COVID‐19 and 30 eyes from 30 sex‐matched healthy controls in Turkey. They used a manual caliper to measure the thickness of the blood vessel wall using infrared images. They found that the average thickness of the vein and artery wall during the active period was significantly higher than that after recovery (*p* = 0.023, *p* = 0.013). However, there was no significant difference between the vascular wall thickness after recovery and the control group. Vascular wall thickening may indicate inflammation. There was no difference between the recovered vascular wall and the control group, indicating that the vascular wall thickening caused by COVID‐19 is reversible [39]. Guemes‐Villahoz et al. studied 90 patients with COVID‐19 in Spain from March to May 2020. All patients underwent optical coherence tomography angiography (OCTA) three months after their COVID‐19 diagnosis. The results showed that the VD of the central, inner, and outer rings in patients with COVID‐19 was significantly lower than that in the healthy control group. The perfusion density of the outer ring and the whole area was also significantly reduced. Fundus manifestations show little correlation with thrombosis formation [40]. However, another study noted that the decrease in central VD and perfusion density after the acute phase of COVID‐19 was significantly related to the increase in D‐dimer levels. Microvascular involvement may be closely related to the increase in D‐dimer levels in patients, that is, the procoagulant state [41].

**Figure 3:**
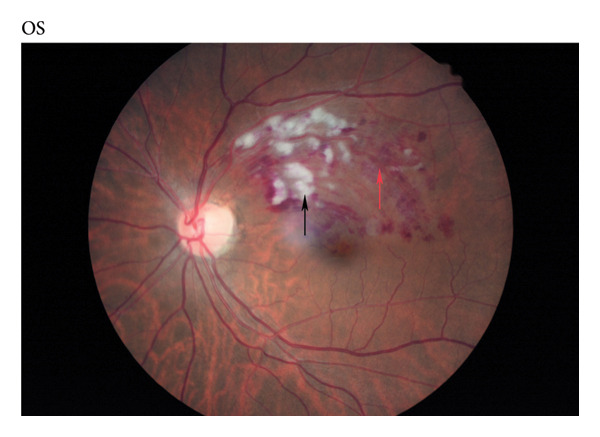
Fundus image of patient with retinal vein occlusion after COVID‐19 infection. A 51‐year‐old male patient whose vision in the left eye dropped to 0.1 a week after infection with COVID‐19. Obstruction of the superior temporal branch vein, hemorrhage in the drainage area (red arrow), retinal edema, and cotton wool spots (black arrow) were observed.

Retinal thrombosis is also an important part of retinopathy caused by COVID‐19. The mechanism may involve COVID‐19 invading cells through ACE2 receptors and activating the renin‐angiotensin system (RAS), leading to an increase in angiotensin II, which causes vasoconstriction and an increase of platelets and white blood cells, thus inducing thrombosis; Another possible mechanism is that COVID‐19 infiltration can lead to retinal vasculitis, rupture of the blood retinal barrier, and retinal degeneration, with red blood cells and immune cells infiltrating surrounding tissues. This interstitial inflammation may lead to vasculitis, resulting in transparent thrombosis [5, 6]. Modjtahedi et al. compared the incidence of retinal embolism in COVID‐19 patients diagnosed in Southern California from January 20, 2020, to May 31, 2021. They compared the incidence of retinal vascular embolism (retinal artery and vein embolism) in 432,515 COVID‐19 patients 6 months before and after diagnosis. Within 6 months after COVID‐19 was diagnosed, 16 patients had retinal artery occlusion (RAO) (3.00 cases per 1 million patients), and 65 patients had retinal vein occlusion (RVO) (12.20 cases per 1 million patients). After COVID‐19 diagnosis, the incidence rate of retinal artery occlusion (RAOS) increased slightly (IRR, 1.35; 95% CI, 0.64–2.85; *p* = 0.44). However, the incidence of RVO increased more significantly (IRR, 1.54; 95% CI, 1.05–2.26; *p* = 0.03). The peak incidence of RAOS and RVOS occurred at 10–12 weeks and 6–8 weeks after diagnosis, respectively. COVID‐19 infection may increase the risk of retinal vascular embolism, especially retinal vein embolism [42]. The fundus picture of a patient with BRVO related to COVID‐19 is shown in Figure 3. In addition, COVID‐19 infection may also lead to papillophlebitis [43]. It is unclear whether COVID‐19 can infect the retina and choroid. Animal experiments have shown that the virus spreads from the lungs to the brain and eyes via a network of olfactory and optic nerves, leading to the ocular manifestations and retinal inflammation [44]. Jidigam et al. compared the pathological sections of 7 COVID‐19‐positive eyes and 6 age‐matched COVID‐19‐negative eyes. They found that SARS‐CoV‐2 spike protein immunoreactivity showed distinct and specific localization in round cells within the retina of all the COVID‐19 eyes near the optic nerve head. Both the ACE2 receptor and the TMPRSS2 protein were present in retinal tissue. Compared to the control group, the retinal microvascular system of COVID‐19 eyes showed changes, with increased inflammation and glial hyperplasia, suggesting that COVID‐19 might directly infect the posterior segment of the eye [45]. Marinho et al. conducted OCT in 12 COVID‐19 patients within 11–33 days after infection and found that all patients’ eyes showed hyperreflective lesions in ganglion cells and the inner plexiform layer, especially in the macular bundle of the optic disc. The OCT angiography and ganglion cell complex analysis results were normal. Four patients exhibited tiny cotton spots and microbleeds along the retinal arch during fundus examination [46]. It is still unclear whether these changes were caused by direct infection of COVID‐19 or secondary changes caused by COVID‐19. Due to the lack of relevant research, it is still impossible to determine whether COVID‐19 can directly infect the retina.

The choroidal thickness increased after COVID‐19 infection, and this change was reversible. Gündoğan compared choroidal thickness changes in 30 eyes of 30 patients with COVID‐19 infection and 30 eyes from a control group in Turkey between December 2020 and May 2021. They found that the choroidal thickness under the fovea, on the nasal side, and on the temporal side was significantly higher than in the control group. Compared to the control group after recovery from COVID‐19, choroidal thickness in all areas was higher, but there was no statistical difference. This suggests that the choroidal thickness increased during COVID‐19 infection but decreased after recovery (Table 1) [39].

**Table 1:** Changes of retina and choroid associated with COVID‐19.

		Sample size	Method	Result	Conclusion	Reference
COVID‐19‐associated retinopathy	Indirect effect	32 COVID‐19 patients (59 eyes)	Fundus photos were taken within 1 month after COVID‐19 infection and follow‐up 6 months (±30 days)	1. At the initial stage, 12 eyes (20.3%) had retinal hemorrhage, 2 eyes had cotton wool spots (3.4%), 12 eyes (20.3%) had venous dilatation, and 10 eyes (16.9%) had venous tortuosity 2. In nonsevere cases, there was no significant difference in mean artery diameter (MAD) and mean vein diameter (MVD) between the follow‐up group and the control group without COVID‐19 infection. However, in severe cases, there was still a significant difference in MAD and MVD between the follow‐up group and the control group without COVID‐19 infection.	Mean arterial diameter and mean venous diameter increase. Retinal vascular changes in nonsevere cases will subside over time, while severe cases cause damage to the vascular system and may lead to long‐term, continuous changes.	[37]
		30 eyes of COVID‐19 patients	Use a manual caliper to measure the thickness of the blood vessel wall of COVID‐19 patients after infection and follow‐up 3 months through infrared imaging	The average thickness of the vein wall and artery wall during the active period was significantly higher than that after recovery (*p* = 0.023, *p* = 0.013). However, there was no significant difference between the recovered vascular wall thickness and the control group	Vascular wall thickness increases. The thickening caused by COVID‐19 is reversible.	[39]
		90 COVID‐19 patients	OCTA was performed 3 months after the diagnosis of COVID‐19	The vascular density of the central, inner, and outer rings in patients with COVID‐19 was significantly lower than that in the healthy control group. The perfusion density of the outer ring and the whole area was also significantly reduced.	Vascular density decreases. Compared to healthy controls, SARS‐CoV‐2‐infected patients have lower vascular and perfusion density.	[40]
		432515 COVID‐19 patients	Incidence rate of retinal vascular embolism (retinal artery embolism, retinal vein embolism) in 432515 patients infected with COVID‐19 6 months before and after diagnosis	After COVID‐19 diagnosis, the incidence rate of retinal artery occlusion (RAOS) increased slightly (*p* = 0.44), while the incidence rate of RVO increased more, and the incidence of retinal vein occlusion (RVOS) increased (*p* = 0.03). The peak incidence of RAOS and RVOS occurred at 10–12 weeks and 6–8 weeks after diagnosis, respectively.	The incidence of vascular embolism increases. COVID‐19 infection may elevate the risk of retinal vascular embolism, especially retinal vein embolism.	[42]
	Direct infection	7 COVID‐19 patients	Pathological section analysis of eyes from 7 COVID‐19‐positive and 6 age‐matched control donors	1. The ACE2 receptor and TMPRSS2 protein are both present in retinal tissue 2. Compared with the control group, the retinal microvascular system of COVID‐19 eyes changed, inflammation increased, and glial hyperplasia	Pathology suggests the presence of COVID‐19 targets and retinal inflammation, indicating that COVID‐19 might directly infect the posterior segment of the eye.	[45]
		12 COVID‐19 patients	OCT was performed between 11 and 33 days after COVID‐19 infection	All patients’ eyes showed hyperreflexic lesions of ganglion cells and the inner plexiform layer, especially in the macular bundle of the optic papilla.	OCT indicates hyperreflective lesions in ganglion cells and the inner plexiform layer, with changes occurring in both after COVID‐19 infection.	[46]

COVID‐19‐associated choroidal lesions		30 COVID‐19 patients (30 eyes)	OCT was performed within 1 week after COVID‐19 infection	Horoidal thickness under the fovea, nasal side, and temporal side was significantly higher than that in the control group. Compared with the control group after the recovery from COVID‐19, although the choroidal thickness in all areas was higher than that in the control group, there was no statistical difference	The choroidal thickness increases after COVID‐19 infection and is reversible, with an increase during infection and a decrease after recovery.	[39]

Abbreviations: ACE2 = angiotensin‐converting enzyme II, MAD = mean artery diameter, MVD = mean vein diameter, OCT = optical coherence tomography, OCTA = optical coherence tomography angiography, RAOS = retinal artery occlusion, RVOS = retinal vein occlusion, TMPRSS2 = transmembrane serine protease.

Another retinal infection is subretinal aspergillus abscess, which is secondary to steroid treatment after COVID‐19 infection. Sahu et al. reported five cases of subretinal Aspergillus abscess caused by COVID‐19 between May 15 and June 16, 2021, in India. All five cases had received systemic corticosteroids for a mean duration of 7 days during severe COVID‐19 treatment. Within 1–31 days after recovery from COVID‐19, patients showed a decline in vision. After cultivation, it was confirmed that there were two cases of *Aspergillus niger* and two cases of *Aspergillus fumigatu*s. Four eyes underwent pars plana vitrectomy with silicone oil injection and showed satisfactory anatomical outcome with control of the infection. However, no significant visual gain was achieved [47].

In general, microangiopathies associated with COVID‐19 include retinal tamponade, retinal hemorrhage, and dilation of retinal veins and arteries. Other changes include increased vessel wall thickness and decreased retinal macular VD. After infection with COVID‐19, the risk of retinal vascular embolism increases, and the risk of retinal vein embolism is higher than that of artery embolism. Unlike diabetic retinopathy, most of these microangiopathies have a better prognosis. The mechanism may be related to microvascular injury or thrombosis caused by the hypercoagulable state of patients and respiratory distress or acute increase in blood pressure, leading to diffuse or focal vasospasm. The presence of COVID‐19 binding sites was found in the eyes of patients who died of COVID‐19, and inflammatory cell infiltration was also seen in the retina. It is suggested that COVID‐19 may directly infect the retina. COVID‐19 patients exhibited significant reflex changes in ganglion cells and the inner plexiform layer, which may be changes caused by COVID‐19 infection of the retina. After infection with COVID‐19, the choroidal thickness increased, but this was a reversible change.

### 3.3. Neuro‐Ophthalmic

#### 3.3.1. COVID‐19‐Related Neuro‐Ophthalmic Diseases

**Figure 4:**
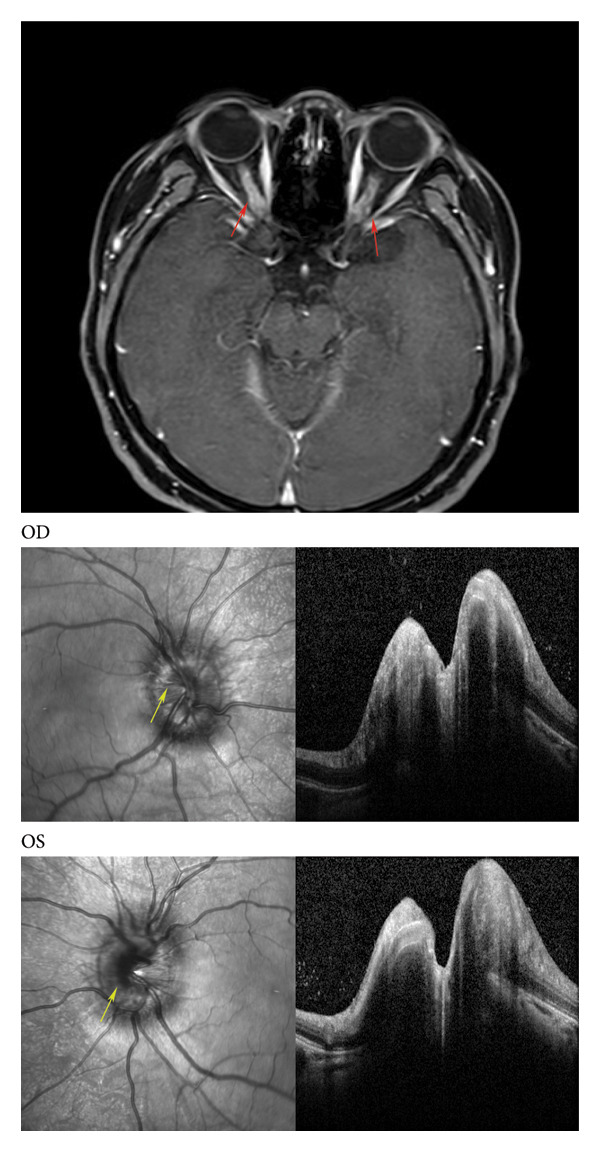
Magnetic resonance imaging and optical coherence tomography of patients with optic neuritis after COVID‐19 infection. A 23‐year‐old female patient complained of right eye pain and decreased vision after infection with COVID‐19. Her vision was 0.08 and 0.5 in the right and left eyes, respectively. Magnetic resonance imaging in T1‐weighted imaging fat‐sat + C sag showed that the optic nerve margins were significantly strengthened (red arrow). OCT showed swollen optic discs in both eyes with blurred borders (yellow arrow). (OD: right eye, OS: left eye).

Neuro‐ophthalmic manifestations in patients with COVID‐19 are rare and usually appear in case reports. COVID‐19 can invade peripheral nerve endings and retrograde propagate through axonal transport, leading to the occurrence of neuropathic eye diseases [7]. COVID‐19‐related afferent neuropathies mainly include optic neuritis, papillophlebitis, papilledema, visual disturbance secondary to posterior reversible encephalopathy syndrome, and loss of vision after stroke. COVID‐19‐related efferent neuropathies mainly include diplopia due to sixth cranial nerve paralysis, ophthalmoplegia caused by Miller Fisher syndrome, ptosis caused by myasthenia gravis, Adie’s pupils, nystagmus, and eye movement disorders [48, 49]. The magnetic resonance imaging and OCT images of a patient with optic neuritis associated with COVID‐19, which leads to papilledema, are shown in Figure 4.

### 3.4. Orbital

#### 3.4.1. ROCM‐Related COVID‐19

Mucor disease is an opportunistic infection caused by Mucor, which causes infarction and tissue necrosis by invading blood vessels, often secondary to diabetes, immune deficiency, and the use of systemic corticosteroids. Since the prevalence of COVID‐19, the widespread use of steroids has led to an increase in the incidence of mucormycosis. On a global scale, the incidence of mucormycosis ranges from 0.005 to 1.7 people per million population, while the incidence in India was nearly 80 times higher between 2019 and 2020 (0.14 people per 1000 people) [50]. The most common type of mucormycosis is ROCM, which is caused by the residence, germination, and further infiltration of spore tubes in the paranasal sinuses into the eye sockets, cavernous sinuses, and brain [51, 52]. The proportion of diabetes, steroid use, and eye involvement in ROCM patients is about 80%–98%, 50%–100%, and 50%–92%, respectively. The most common symptoms were facial pain and swelling, and the most common sign was ptosis. The proportion of bilateral involvement is relatively low, generally less than 10%. The interval between COVID‐19 and ROCM diagnosis is about 14–25 days. About 56% of patients were diagnosed within 14 days, while 44% of patients were diagnosed after 14 days [53]. In another study, 26% of patients were diagnosed during hospitalization and 74% after discharge [54]. Intravenous injection of amphotericin B and sinus debridement are routine treatment measures, while other treatment measures include oral posaconazole/esoconazole, enucleation of the eyeball, and mandibular resection. Systemic steroid use and diabetes history are the risk factors for ROCM in COVID‐19 patients. The mortality rate is about 5%–10%, the incidence of enucleation is between 10% and 16%, and the visual prognosis is poor. Ophthalmology interventions to reduce mortality rates mainly include eye enucleation for the patients with ROCM Stages 3 and 4, and retrobulbar injection of amphotericin B may also reduce mortality rates (Tables 2 and 3) [51, 53–55].

**Table 2:** Clinical features of COVID‐19–associated ROCM.

Reference	Date	Locality	Patient number	Age	Gender	Severity of COVID‐19 *n* (%)	Combined diabetes *n* (%)	Combined the use of steroids *n* (%)	Eye involvement *n* (%)	Common symptoms *n* (%)	Common signs *n* (%)	Bilateral involvement	Common species
[53]	2020.1.1–2021.5.26	Various regions in India	2826 COVID‐19 patients	51.9	Male 71% Female 29%	*n* = 2818 Asymptomatic 54 (1.9%) Home care 735 (26%) Hospitalized 2029 (72%)	*n* = 2825 2194 (78%)	*n* = 2371 2073 (87%)	1917 (72%)	Orbital/facial pain (23%) Orbital/facial edema (21%) Loss of vision (19%) Ptosis (11%) Nasal block (9%)	Periocular/facial edema (33%) Loss of vision (21%) Ptosis (12%) Proptosis (11%) Nasal discharge (10%)	230 (8.6%)	—

[50]	2021.2.1–2021.5.10	Six centers of India	38/1546 COVID‐19 patients	54	Male 68% Female 32%	Oxygen supplementation at admission 25 (66%) Non‐invasive ventilatory support initiated within 24 h of admission 7 (18%) Invasive ventilation 6 (16%)	19 (50%)	38 (100%)	21 (55%)		Ptosis 19 (90%) Ophthalmoplegia 18 (47%) Total vision loss 15 (39%) Orbital edema 11 (29%)	None	Culture and species identification were carried out in 23 patients (69.7%). Rhizopus (60.9%) Mucor (21.7%) Absidia (17.4%)

[51]	2021.9.1–2021.12.31	Eight centers of Iran	274	56.8	Male 55% Female 45%	Outpatient 56 (20.4%) Inpatient 218 (79.6%) Ward 195 (89.4%) ICU (Intensive care unit) 23 (10.6%) Lung involvement severity Mild 100 (36.5%) Moderate 159 (58%) Severe 15 (5.5%)	227 (82.8%)	202 (73.7%)	253 (92.3%)	Facial pain (47.4%) Facial swelling (38.3%) Nasal discharge (32.5%)	Ptosis (58%) Periorbital swelling (46%) Nasal congestion (40.5%)	17 (6.7%)	—

[52]	2021.5–2021.9	East India	219	49.9	Male 69% Female 31%	—	90 (81.8%)	56 (50.9%)		Unrelenting ocular or facial pain was the most common presenting symptom 69 (31.5%)	Ptosis was the most common anterior segment manifestation. 51 (23.2%) Ophthalmic artery occlusion was the most common posterior segment manifestation 21 (9.6%)		—

[55]	2021.5.15–2021.6.5	India	60	57 (median age)	Male 75% Female 25%	Mild: 26 (45.6%) Moderate: 28 (49.1%) Severe: 3 (5.3%)	59 (98.3%)	38 (63.3%)	35 (58.3%)	Facial pain was the commonest first symptom of ROCM patients.	Ptosis 29 (48.3%) Total ophthalmoplegia 18 (30%) Periocular tenderness 17 (28.3%) Periocular edema 16 (26.6%) Proptosis 14 (23.3%) Diminution of vision 12 (20%) Complete loss of vision 10 (16.6%) Central/branch retinal artery occlusion 8 (13.3%)		—

Abbreviations: ICU = intensive care unit, ROCM = rhino‐orbito‐cerebral mucormycosis.

**Table 3:** Diagnosis, treatment, and prognostic outcomes of COVID‐19–associated ROCM.

Reference	Diagnosis time *n* (%)	Treatment plan *n* (%)	Prognosis *n* (%)	Prognostic factors
[53]	The mean interval was 14.5 ± 10 days (*n* = 2285, median 13 days), with 56% of the patients developing within 14 days. Delayed manifestation after 14 days was seen in 44%.	Intravenous amphotericin B 2066 (73%) *n* = 2066 Oral posaconazole/ isavuconazole 732 (25.9%) *n* = 732 Intravenous amphotericin B + oral posaconazole/isavuconazole 657 (23.2%) *n* = 657 FESS/Paranasal sinus debridement 1585 (67%) *n* = 2358 Orbital exenteration 339 (15%) *n* = 2327 FESS/Paranasal sinus debridement + orbital exenteration 367 (17%) *n* = 2186	*n* = 2218 41% (909) were alive and well with regression of ROCM 32% (717) were alive with clinico‐radiologically stable ROCM 13% (287) had progressive ROCM on treatment 14% (305) had expired. *n* = 1838 Orbital exenteration 289 (16%) Eye salvage 1549 (84%) *n* = 1426 PL negative 298 (21%) PR inaccurate 88 (6.2%) ≤ 20/200 195 (14%) 20/100–20/40 317 (22%) ≥ 20/40 528 (37%)	Orbital exenteration significantly alters the outcome in patients with disease Stages 3 and 4 (*p* < 0.05)

[50]	The median time to diagnosis of ROCM from the day of hospital admission was 17.5 (12–22) days. 10 (26%) diagnosis of ROCM during hospitalization 8 (21%) diagnosis of ROCM within a week of discharge 15 (39.5%) diagnosis of ROCM within 2 weeks of discharge 5 (13%) diagnosis of ROCM within a month of discharge	All patients with an established ROCM diagnosis were treated with intravenous amphotericin B (deoxycholate‐23, liposomal‐15) with a median dose of 0.9 (0.3–1.0) mg/kg/day over a median duration of 21.5 days (14–28) 17 (45%) received additional therapy with oral posaconazole 27 (71%) endoscopic debridement 6 (16%) orbital exenterations 3 (8%) hemi‐mandibulectomy 5 (13%) medical therapy alone	Death 2 (5%) Orbital exenteration 6 (16%)	

[51]	The duration from COVID‐19 infection to the CAM diagnosis was 25.2 ± 30.22 days; the median time was 19 days.	FESS/sino‐nasal debridement 152 (55%) FESS/sino‐nasal debridement + TRAMB injection 94 (34.3%) FESS/sino‐nasal debridement + orbital exenteration 28 (10.2%) All patients with an established ROCM diagnosis were treated with intravenous amphotericin B	30 (10.9%) deaths 28 (10.2%) orbital exenteration The mean LogMAR best corrected visual acuity (BCVA) of the affected eyes was 2.77 ± 2.02. Extraocular movement restriction and the frozen eye were developed in 72.8% of eyes. 63 (23.3%) and 10 (3.7%) of the affected eyes had atrophic discs and optic disc swelling, respectively. CA‐ROCM‐associated microvascular event, and branch retinal vein occlusion (BRVO) was present in 45 eyes	FESS/sino‐nasal debridement + TRAMB injection can significantly reduce the risk of patient mortality. Risk factors for death: History of diabetes (*p* = 0.01), new‐onset diabetes (*p* = 0.003), smoke (*p* = 0.02, positive SARS‐CoV‐2 RT‐PCR test (*p* = 0.002), admission to ICU (*p* = 0.006), systemic corticosteroids (*p* = 0.003), the stage of CAM (*p* < 0.001), orbital exenteration (*p* = 0.005), TRAMB treatment (*p* = 0.004), bilateral ocular involvement (*p* = 0.003)

[52]	Mean ± SD interval of 20.9 ± 12.6 days	—	The presenting visual acuity was equal to or better than 6/60 in 147 eyes (66.8%) 34 eyes had no perception of light 10 eyes had perception of light only without being able to perceive hand movements.	—

[55]	The median duration was 17 days	All the patients were treated with IV amphotericin B liposomal. Functional endoscopic sinus surgery (FESS) was performed in 10 patients. In 2 cases FESS was combined with maxillectomy and orbital exenteration. The remaining 48 patients were planned for radical sinus debridement.	—	—

Abbreviations: CAM = COVID‐19 associated mucormycosis, FESS = functional endoscopic sinus surgery, ICU = intensive care unit, PL = light perception, ROCM = rhino‐orbito‐cerebral mucormycosis, RT‐PCR = reverse transcription‐polymerase chain reaction, TRAMB = transcutaneous retrobulbar amphotericin B.

### 3.5. Tears and Conjunctival COVID‐19 Detection in the Diagnosis of Eye Lesions

As COVID‐19 infection can affect the eyes, some studies have explored the role of nucleic acid detection in tears and conjunctival swabs in diagnosis. Azzolini et al. tested the conjunctival swabs of 91 severe COVID‐19 patients from three intensive care units (ICUs) in Italy between April and May 2020. In addition, 57% (51 patients) tested positive for conjunctival swabs, including 17 patients with ocular manifestations. Conjunctival swabs of 40 patients were negative, including 2 patients with ocular manifestations [56]. In this study, positive conjunctival swabs were related to the occurrence of ocular manifestations. In fact, a large number of studies have noted that the results of conjunctival swabs have little correlation with COVID‐19‐related eye manifestations [3, 57–63]. Cao et al. summarized 12 studies including 1930 patients with COVID‐19 through meta‐analysis from December 2019 to September 2020, and the positive rate of conjunctival swabs was 3%. Their study suggested that positive conjunctival swabs were not related to ocular manifestations. Therefore, more research is still needed to determine whether conjunctival swabs can help to detect ocular manifestations related to COVID‐19 [64].

### 3.6. COVID‐19 Vaccine and Eye Diseases

With the extensive vaccination for COVID‐19, some new and recurrent eye diseases after COVID‐19 vaccination have attracted the attention of ophthalmologists. The incidence of COVID‐19 vaccine‐related eye diseases is low, and they are usually mild and reversible [65]. However, since most people are vaccinated against COVID‐19, we still cannot ignore eye diseases that may be related to the COVID‐19 vaccine. The meta‐analysis conducted by Yu in August 2021 summarized 31 studies on eye manifestations after COVID‐19 vaccine injection, including lesions in eyelids, cornea and ocular surface, retina, uvea, nerves, vascular thrombosis, and other parts. There were 6 cases of ocular manifestations on the eyelids, including eyelid swelling, herpes zoster ophthalmicus (HZO), and eyelid purpura lesions. Six cases occurred in the cornea and ocular surface (corneal graft rejection after penetrating keratoplasty [PKP] and corneal graft rejection after Descemet membrane endothelial keratoplasty), 7 cases of ocular manifestations in the uvea (acute anterior uveitis, panuveitis, acute local occult external retinopathy [AZOOR], multifocal choroiditis, VKH disease, and uveitis), 7 cases of ocular manifestations in the retina (acute macular neuropathy [AMN], central serous retinopathy, and retinal detachment), 3 cases of ocular manifestations of nerves (optic neuritis, arterial preischaemic optic neuropathy [AAION], and abrupt nerve paralysis), and 8 cases of ocular manifestations with thrombosis (superior ophthalmic vein [SOV] thrombosis, cerebral venous sinus thrombosis [CVST], and thrombocytopenia with acute ischemic stroke and hemorrhage) [66].

The most common ocular adverse reaction after COVID‐19 vaccine injection is uveitis, which may be caused by the immune response to vaccine adjuvants, molecular mimicry between vaccine peptide fragments and uveal self‐peptides, and delayed hypersensitivity and subsequent immune complex deposition as the potential causes. The most common type of uveitis after COVID‐19 vaccine injection is anterior uveitis. Testi et al. summarized 70 patients with ocular inflammation within 14 days after the injection of the COVID‐19 vaccine collected in 40 centers around the world in March 2021, revealing the specific incidence of various types of uveitis after the injection of the COVID‐19 vaccine. Among all patients, 36 (54.1%) had a history of ocular inflammation, including 28 patients who had been under stable control for more than 3 months, 7 patients who had received systemic anti‐inflammatory treatment, and 1 patient who had received local anti‐inflammatory treatment. These patients did not stop or change their medication due to the injection of the COVID‐19 vaccine. In this study, 41 (58.6%) patients developed anterior uveitis after COVID‐19 vaccination, followed by posterior uveitis (9 cases, 12.9%), total uveitis (3 cases, 4%), and intermediate uveitis (2 cases, 3%). It is worth noting that the disease situation of patients with a previous history of ocular inflammation after vaccination is similar to the previous history, and most of the inflammation is also mild, with a good prognosis [67]. Mahendradas et al. summarized 67 patients (98 eyes) who had ocular inflammation within 8 weeks after COVID‐19 vaccination in 2021 in India and found similar results. The most common one was anterior uveitis (*n* = 31, 31.7%), followed by panuveitis (*n* = 24, 24.5%). Among 67 patients, 39 patients (58.2%) had a history of ocular inflammation [68]. However, a study has pointed out that the most common ocular inflammation associated with the COVID‐19 vaccine is VKH, which may be related to the relatively high incidence rate of VKH in Asia [69].

However, a recent study between August 9, 2021 and May 31, 2022 in Beijing, China, pointed out that compared with 60 patients with uveitis who were not vaccinated with the COVID‐19 vaccine, the incidence of flares in 60 patients who were vaccinated with the inactivated COVID‐19 vaccine did not increase at 30 and 60 days of follow‐up. They believed that the recurrence of uveitis might not occur in patients with uveitis who were vaccinated with inactivated COVID‐19 vaccine. However, whether the COVID‐19 vaccine will lead to recurrence of uveitis or whether some type of COVID‐19 vaccine will lead to recurrence of uveitis is still controversial [70]. In addition, uveitis after COVID‐19 vaccination is not unusual. Almost all vaccines currently in use have reported vaccine‐related uveitis cases, including hepatitis A and B virus, human papillomavirus, influenza virus, and measles–mumps–rubella vaccines, so new and recurrent uveitis after vaccination is not specific to COVID‐19 vaccination [71–74].

Most of the retinal adverse events after COVID‐19 were retinal artery and vein occlusions. The main cause of thrombosis after vaccination may be thrombocytopenia, also known as vaccine‐induced immune thrombotic thrombocytopenia (VITT). (Risk assessment of retinal vascular occlusion after COVID‐19 vaccination.) From May 2021 to January 2022, Choi et al. included 16 patients with ocular manifestations within 1 week after COVID‐19 vaccination in Korea. In these patients, RVO was found in 9 eyes (52.9%), retinal artery occlusion was found in 1 eye (5.9%), new anterior uveitis was found in 1 eye (5.9%), aggravation of previously diagnosed panuveitis was found in 2 eyes (11.8%), and closure attack of high intraocular pressure was found in 4 eyes (23.5%) [75]. Retinal vascular occlusion is one of the adverse eye reactions to the COVID‐19 vaccine. Based on the data provided by the TriNetX global network, Li et al. analyzed the data collected from American patients from January 1, 2020, to December 31, 2022. They excluded individuals with a history of retinal vascular occlusion or who used any systemic drugs that might affect blood coagulation before vaccination. Compared to the individuals who did not receive the COVID‐19 vaccine, individuals who received the COVID‐19 vaccine had a higher risk of developing various forms of retinal vascular occlusion within 2 years after vaccination, with an overall risk ratio of 2.19 (95% confidence interval 2.00–2.39). The risk of retinal vascular occlusion significantly increases in the first 2 weeks after vaccination and lasts for 12 weeks. However, different vaccine brands and doses are not associated with retinal vascular occlusion [8]. Ruiz and Gonzalez‐Lopez reported a case of branch retinal artery occlusion and central RVO in the left eye 12 days after the second dose of the Moderna Lonza vaccine (an mRNA COVID‐19 vaccine) was inoculated, but the causal relationship between the COVID‐19 vaccine and ocular diseases cannot be determined [76]. (Table 4).

**Table 4:** COVID‐19 vaccine and eye diseases.

Reference	Date	Locality	Sample size	Age	Time of appearance of ocular manifestations following COVID‐19 vaccination	Type of vaccine; symptoms appear after the first second dose	Mean time after dose	Medical history of the eyes	Eye manifestations after COVID‐19 vaccination
[67]	2021	40 centers around the world	70 patients	51	In 2 weeks	Pfizer 40 (57.1%) AstraZeneca 17 (24.3%) Moderna 10 (14.3%) Sinopharm 2 (2.9%) Covaxin 1 (1.4%) First dose 43 (61.4%) Second dose 27 (39.6%)	First dose 6 days Second dose 5 days	36 (54.1%) had a history of ocular inflammation 28 patients had been under stable control for more than 3 months 7 patients had received systemic anti‐inflammatory treatment. 1 patient had received local anti‐inflammatory treatment.	Anterior uveitis 41 (58.6%) Posterior uveitis 9 (12.9%) Total uveitis 3 (4%) Intermediate uveitis 2 (3%) Panuveitis 3 (4.3%) Optic neuritis 2 (2.9%) Episcleritis 2 (2.9%) Intermediate uveitis 2 (2.9%) Paracentral acute middle maculopathy 1 (1.4%) Giant cell arteritis 1 (1.4%) Periocular skin herpes zoster 1 (1.4%) Unspecific blurriness of vision 1 (1.4%)

[68]	2021	South India	67 patients (98 eyes)	43	In 8 weeks	COVISHIELD™ vaccine 56 (83.6%) (31 (55.2%) first dose; 28 (41.8%) second dose; 2 (3%) both first and second doses) Covaxin vaccines 9 (13.4%) Sputnik vaccines 1 (1.5%) Pfizer vaccines 1 (1.5%)	25 days	39 (58.2%) patients with a formerly diagnosed uveitis	Anterior uveitis 31 eyes of 26 patients (31.7%) Panuveitis 24 eyes of 13 patients (24.5%) Posterior uveitis 12 eyes of 8 patients (12.2%) Episcleritis 10 eyes (10.2%) Keratouveitis 4 eyes (4.1%) Sclerokeratouveitis 4 eyes (4.1%) Scleritis 1 eye (1%)

[75]	2021–2022	Korea	16 patients (17 eyes)	63.8	In 1 week	AstraZeneca vaccine 12 (75%) Pfizer vaccine 4 (25%) First dose 10 (58.8%) Second dose 5 (29.4%) Third dose 2 (11.8%)	3.65 days	—	Retinal vein occlusion 9 (52.9%) retinal artery occlusion 1 (5.9%) Newly developed anterior uveitis 1 (5.9%) Exacerbation of previously diagnosed panuveitis 2 (11.8%) Angle‐closure attack with high intraocular pressure 4 (23.5%)

It is unclear whether the incidence of eye diseases is related to the type of vaccine. We summarized 193 patients with adverse eye reactions within one month after the injection of the COVID‐19 vaccine and counted the types of vaccines they received. The results showed that the vaccines injected by patients with ocular adverse reactions were mainly nucleic acid vaccines (74%, BNT162b2 mRNA), followed by viral vector vaccines (16%), attenuated inactivated vaccines (7%), and subunit vaccines (4%). The largest proportion of nucleic acid vaccines in eye adverse events does not mean that nucleic acid vaccines are the most likely to cause eye adverse events but more likely because nucleic acid vaccines are the main vaccination vaccines in some countries. Regarding whether a certain type of vaccine is more likely to cause adverse eye reactions, more research is still needed [67, 77, 78].

There are many differences in mechanism, incidence rate, type, and prognosis between ocular manifestations caused by the COVID‐19 infection and COVID‐19 vaccine. The main mechanisms of ocular manifestations caused by the COVID‐19 infection are direct infection, cytokine storm, hypercoagulability, and the immunity caused by excessive use of steroids. For ocular manifestations caused by the COVID‐19 vaccine, mechanisms include changes in immune status and vaccine‐induced immune thrombotic thrombocytopenia. The incidence rate of ocular manifestations caused by the COVID‐19 infection is about 11%, including ocular foreign body sensation, conjunctival congestion, epiphora, and conjunctival secretion. Eye diseases caused by COVID‐19 infection include conjunctivitis, with the incidence rate in COVID‐19 patients is about ranging from 0.9% to 36%. Its symptoms are mild and are self‐limited. The incidence of uveitis caused by COVID‐19 is low. After infection with COVID‐19, the incidence rate of RAOS is slightly higher, and the incidence of vein occlusion is higher than artery occlusion. During the COVID‐19 pandemic, the incidence rate of ROCM in India (0.14/1000 people) is about 80 times higher than the global incidence. COVID‐19 infection can also lead to retinal infection and optic neuropathy. The incidence of ocular manifestations caused by the COVID‐19 vaccine is low and very mild, and uveitis is the most common manifestation. Within 2 years after vaccination, the risk of retinal obstruction is higher, with an overall risk ratio of 2.19 [5–8].

## 4. Conclusion

Various studies have shown that the eyes may be one of the entry points of the COVID‐19 virus. COVID‐19‐related ocular manifestations are mainly conjunctival congestion and dry eyes. Patients with severe pneumonia may be more likely to have ocular manifestations. There are also cases involving the uvea and retina. The infection rate and hospitalization rate of patients with uveitis were not different from those of the control group. ROCM has a high mortality rate and should be identified early and treated promptly. Conjunctival swabs have little significance for the detection of eye manifestations related to COVID‐19. The incidence of COVID‐19 vaccine‐related ocular manifestations is low and may involve all parts of the eye, and the disease is mild and reversible. Conjunctivitis, the main ocular manifestation of patients with COVID‐19 infection, is not common in COVID‐19 vaccine‐related ocular diseases. It may differ depending on the substances entering the body (live virus and various vaccines) or the ways of entering the body (respiratory tract, direct contact, and intramuscular injection). However, the specific mechanism still needs to be explored in subsequent studies. This study suggests that we should pay more attention to the eye changes of patients with COVID‐19, especially ROCM, which may lead to death. In case of ocular manifestations, we should pay attention not only to the changes of the eye surface but also to the fundus lesions. After the injection of the COVID‐19 vaccine, it is also necessary to pay attention to the possibility of uveitis and retinal thrombosis. In long COVID, more research is needed to focus on the long‐term effects of COVID‐19 on the eyes.

## Disclosure

All authors agreed to the published version of the manuscript.

## Conflicts of Interest

The authors declare no conflicts of interest.

## Author Contributions

Mengyao Li conceived of and developed the structural design of the article and wrote the manuscript. Tao Liu reviewed the manuscript and contributed significant revisions. Lin Zhu and Ruyu Liang organized the relevant literature, wrote the manuscript, and made important revisions. Peng Zhang organized the relevant literature and made important revisions. Xiaoli Liu conceived the article, improved the layout and structure of the manuscript, and provided suggestions for important revisions. All the authors reviewed and edited the manuscript. Tao Liu and Mengyao Li contributed equally to this manuscript.

## Funding

This work was supported by the National Natural Science Foundation of China, grant no. 81300752; Jilin Province Science and Technology Development Plan Project, grant no. 20200201333JC; and Changchun Science and Technology Development Plan Project, grant no. 21ZGM19.
